# Perspectives of healthcare stakeholders in Nigeria on the impact of COVID-19 on health services

**DOI:** 10.4102/jphia.v16i1.674

**Published:** 2025-01-29

**Authors:** Adeponle O. Adeoye, Yewande Tolulope Nejo, Chinwe Lucia Ochu, Josephine Bayigga, Rodgers Rodriguez Ayebare, Adedayo Omotayo Faneye, Charles Olaosebikan Adewemimo, Oluwaseun Emmanuel Falayi, Adeniyi Francis Fagbamigbe, Prosper Okonkwo, Adewale Victor Opayele, Gloria Ogochukwu Nwiyi, Sunday Obiajunwa Eziechina, Ikemefule Rex Uzoma, Priscilla Ibekwe, Tamrat Shaweno, Nebiyu Dereje, Francis Kakooza, Mosoka Papa Fallah, Georgina Njideka Odaibo

**Affiliations:** 1 Department of Sociology, Faculty of Social Sciences, University of Ibadan, Nigeria; 2 Department of Virology, College of Medicine, University of Ibadan, Nigeria; 3 Nigeria Centre for Disease Control and Prevention, Abuja, Nigeria; 4 Infectious Diseases Institute, Makerere University, Uganda; 5 Department of Epidemiology and Medical Statistics, College of Medicine, University of Ibadan, Nigeria; 6 APIN Public Health Initiative, Abuja, Nigeria; 7 Africa Centres for Disease Control and Prevention, Addis Abada, Ethiopia; 8 African Forum for Research and Education, Kwame Nkrumah University of Science and Technology, Kumasi, Ghana

**Keywords:** COVID-19, service delivery, utilisation, infrastructure, planning

## Abstract

**Background:**

COVID-19 disrupted critical public health services globally. It is important to understand how the pandemic affected healthcare service delivery and utilisation in Nigeria to guide planning for future public health crises in the country.

**Aim:**

This study aimed to explore how the COVID-19 pandemic affected health service delivery and utilisation in Nigeria.

**Setting:**

The study was conducted in Abuja, and Oyo State, Nigeria, in 2023.

**Methods:**

This was a cross-sectional qualitative study that adopted an exploratory study design. Key Informant Interviews were used to elicit information from twenty-eight healthcare stakeholders in relevant government health Ministries and Agencies as well as partners of the government on health. The selection of the stakeholders was done through purposive sampling. Recorded interviews were transcribed verbatim, and analysed using an inductive qualitative data analysis method to generate themes. The data were further organized and analysed using NVivo software version 14.

**Results:**

The findings revealed that COVID-19 negatively affected healthcare service delivery and utilisation in Nigeria. This was due to various factors such as healthcare workers’ unwillingness to provide services, exposed healthcare system gaps that affected service delivery, and the shift of attention and resources to COVID-19. However, the pandemic also presented an opportunity to improve public health infrastructure and health service delivery.

**Conclusion:**

Government needs to maximise the gains from the pandemic to build a resilient health system.

**Contribution:**

This article provides insights for public health policy and planning aimed at enhancing resilience and optimising service delivery during future health crises.

## Background

Coronavirus disease 2019 (COVID-19) is an extremely contagious respiratory illness caused by the virus known as severe acute respiratory syndrome coronavirus 2 (SARS-CoV-2). The pandemic registered a significant increase in morbidity and mortality rates,^1^ with millions infected and over seven million fatalities reported worldwide.^2^ Since the reporting of the first COVID-19 case in Nigeria on 27 February 2020,^3^ over 267 188 cases continued to be reported, with 3155 reported deaths as of 11 February 2024.^2^ In Nigeria, the response to the COVID-19 pandemic was similar to those in other nations. There were border closures, school closures, travel bans, lockdowns and postponements of public activities, among others, as containment and mitigation efforts by the Nigerian government. These efforts have had a notable impact on the nation’s economy, including the healthcare system.^4,5^

In recent years, Nigeria has been managing multiple epidemics including several infectious diseases, such as human immunodeficiency viruses (HIV) and/or acquired immunodeficiency syndrome (AIDS), yellow fever, malaria, meningitis, cholera, anthrax, diphtheria, and Lassa fever.^6,7^ With the COVID-19 pandemic in the picture, countries with limited resources like Nigeria are reported to have experienced substantial disruption in their health service delivery, burdening its already feeble healthcare system.^8^ The pandemic has significantly strained the health systems and the healthcare workforce, resulting in deficiencies in essential pharmaceuticals, medical equipment and human resources. These gaps have made managing other health disorders more difficult.^5^ The pandemic has also exposed the deficiencies in the health system. The pandemic has negatively impacted treatment and prevention services for infectious and non-communicable diseases.^9^

During the pandemic waves, healthcare workers (HCWs) were afraid they would get the virus and spread it to their families, especially after seeing their coworkers get sick.^10^ Patients were also unable to attend acute care visits and follow-up appointments because of fear and anxiety during this time, which caused healthcare facilities to postpone several essential services.^11,12,13^

Research on the COVID-19 pandemic has documented significant shifts in healthcare service operations because of lockdowns and orders to stay home.^14,15^ These modifications include a significant decrease in service delivery, especially in areas severely affected by the pandemic, and some targeted improvements, including telemedicine.^16^ Many individuals were denied access to essential medical care, including cancer treatments that can prolong life or vaccinations.^15,17,18^ Reductions in vital interventions for maternal and child health may result in over a million more child deaths, according to estimates,^19^ and a World Health Organization (WHO) survey indicated that disruptions to healthcare services were most severe in lower-income countries.^20^ It has been stated that in order to inform future public health emergency preparedness, it is necessary to comprehend how COVID-19 affected healthcare systems in various nations.^21^ The purpose of this study was to learn how Nigerian health stakeholders perceived the impact of COVID-19, thereby providing a benchmark for future interventions and effective management of health services in case of future pandemics in the country.

## Research methods and design

### Study design

This was a cross-sectional qualitative study that adopted an exploratory study design.

### Setting

The study was conducted in Nigeria. Nigeria is a country in West Africa with an estimated population of over 200 million.^22^ The country has a high burden of diseases such as malaria and HIV and/or AIDS among other diseases associated with high morbidity and mortality.^23,24^ It is also one of the countries with the highest infant and maternal mortality globally.^25^ Healthcare services in Nigeria are categorised into three levels: primary, secondary and tertiary. The primary healthcare centre is the entry point and the closest to the people providing services like maternal healthcare, infant immunisation and treatment of less serious conditions. The secondary and tertiary healthcare facilities are utilised based on a referral system for complex health conditions. The healthcare systems are managed by federal and state governments through government Ministries and Agencies. Although there are private hospitals established and managed by private individuals and religious bodies in the country, a substantial number of Nigerians still rely on services provided by public healthcare institutions because of the high cost of services provided by private healthcare providers in the country. The study was conducted in Oyo State and Abuja, Nigeria. Oyo State was selected because it was one of the states that reported a high rate of COVID-19 in the country, while most national stakeholders in the health sector operate from Abuja, the Federal Capital Territory (FCT).

### Study population and sampling strategy

Purposive sampling method was adopted to select institutions and departments as well as informants. Government healthcare institutions and departments that qualified for the study were those in charge of hospital administration and essential health services like HIV and/or AIDS, tuberculosis, maternal and child health and pharmaceutical supplies at both federal and state government levels. The institutions include the Federal Ministry of Health, National Primary Health Care Development Agency (NPHCDA), Oyo State Ministry of Health and Oyo State Hospital Management Board and those in charge of public health emergency response such as the Nigeria Centre for Disease Control and Prevention (NCDC). Relevant government partners in healthcare development such as the WHO and United Nations Children’s Fund (UNICEF) were also included. The criteria for selecting the informant were those who have oversight roles in the selected government health institutions, departments or units that can provide crucial information that could benefit the study objective. Such individuals must have held their oversight role for at least a year before the pandemic outbreak in Nigeria and also managed their institution or department during the pandemic. This ensured that the experiences of key informants selected captured the situation of healthcare delivery and utilisation in their institution or department before and during the pandemic.

### Data collection

Key informant interviews (KIIs) were used to elicit information from selected informants. An interview guide was designed to probe the impact of COVID-19 on health services using the WHO checklist for health services.^26^ The questions in the interview guide probed the challenges and opportunities observed in the delivery of healthcare services during the pandemic such as routine healthcare services, maternal and immunisation programmes, access and utilisation of healthcare services by other vulnerable groups such as those with chronic diseases and the elderly, modifications made in health planning and financing in health institutions, and the opportunities that the pandemic offered. Two skilled researchers with competence in behavioural science research and Good Clinical Practice and Human Subjects Protection training conducted the interviews to ensure consistency in both data collection and responsiveness to the study’s objectives. The research assistants were briefed about the study objective and trained on the guide before proceeding with data collection. Interviewers contacted respondents on phone and shared information about the study. Each respondent shared a convenient schedule and venue for the appointment, and all the interviews were face to face. The respondents provided written informed consent including authorisation to have the interview recorded on an audio device. All the interviews were conducted in English language. The average length of all interviews was 30 min. The recorded interviews were randomly listened to by the site supervisor to ensure data quality. The study’s provisional data findings were also presented to other team members for credibility and data quality assessment through debriefing.

### Data analysis

Twenty-eight interviews were transcribed from the audio recording verbatim and reviewed to check for consistency with the audio files. Research in qualitative methodology indicates that a sample size of 20–30 participants is often sufficient for achieving saturation, particularly when the population is well defined and the research questions are specific.^27^ Our decision to include twenty-eight respondents was grounded in these qualitative research principles, allowing us to gather rich and comprehensive data necessary to address our research objectives effectively. This sample size provided a robust basis for our analysis while ensuring that we adequately explored the participants’ experiences and opinions. A coding framework was then developed based on an inductive analysis of eight transcripts selected from different categories of participants interviewed by two coders. The coders achieved consensus through rigorous discussions, arriving at mutually agreed-upon codes and themes within the framework. To enhance the validity and comprehensiveness of the framework, investigators conducted a thorough review, contributing an additional layer of consensus building. The final coding framework was entered in NVivo software version 14, and all transcripts were imported to NVivo for further data categorisation and analysis aligned with the established framework. This approach ensures a reliable foundation for the subsequent stages of analysis. While coding, the new themes were identified and added to the initial coding framework. The consistency of the excerpts categorised was rechecked after coding was completed to ensure that the excerpts were adequately categorised. The most comprehensive excerpts were selected as illustrative quotes to discuss themes, and a final codebook was generated after analysis. The source of qualified quotes for analysis was concealed to ensure anonymity and prevent participant tracing.

### Ethical considerations

Ethical clearance to conduct this study was obtained from the National Health Research Ethics Committee (NHREC) and the Oyo State Ministry of Health Research Ethics Committee (HREC) (No. NHREC/OYOSHRIEC). Written informed consent was obtained from all participants. Investigators and research assistants completed the National Institute of Drug Abuse (NIDA) training on good clinical practice and research ethics.

## Results

This section discusses the key themes and sub-themes explored in the analysis of the KIIs and the frequency of sub-themes as presented in Table 1.

**TABLE 1:** Impact of COVID-19 on healthcare service delivery and utilisation in Nigeria.

Themes	Sub-themes	Frequency of sub-themes discussed
1. Negative impact of COVID-19 on healthcare services and utilisation	1.1 Unwillingness of healthcare workers to deliver services	14
	1.2 Exposed health system gaps – inadequate PPEs	8
	1.3 Attention and resources shifted from other diseases to COVID-19	10
	1.4 Disruption in the supply chain of essential drugs	5
	1.5 Restriction of movement affected healthcare delivery and utilisation	4
	1.6 Infection among HCWs disrupted services	3
	1.7 Fear of healthcare utilisation among patients	10
2. Positive impacts of COVID-19 on health service delivery and utilisation	2.1 Improved public healthcare infrastructure	25
	2.2 Introduction of telemedicine in some hospitals	3
	2.3 Provided structure that can be leveraged for future health crises in some states	4
	2.4 Readily trained health workforce for future health emergencies	7

COVID-19, coronavirus disease 2019; HCW, healthcare worker; PPE, personal protective equipment.

### Theme 1: Negative impacts of COVID-19 on healthcare services and utilisation

Participants discussed various ways through which COVID-19 negatively affected healthcare service delivery and utilisation in Nigeria. These discussions are organised into the following sub-themes.

#### Sub-theme 1.1: Unwillingness of healthcare workers to deliver services

Unwillingness of healthcare workers to deliver healthcare services at the early phase of the pandemic was the most discussed theme among the participants regarding the negative impact of COVID-19 on healthcare delivery. Most of the participants mentioned that HCWs were unwilling to deliver services to the people in the early phase of the pandemic because of the fear of contracting the disease, which resulted in cases where some HCWs stayed away from the hospital or did not even attend to patients who presented themselves in some health facilities, resulting in unavailability of healthcare services for patients who needed care. One of the participants mentioned:

‘Before the pandemic, we had a normal program flow, there was no problem with it. We go out to do sensitisation in communities, we do a lot of advocacy then our diagnostics all over. So, there was no, there was no serious challenge, but immediately the COVID came, health workers refused to come to work yes, because they were scared to contract COVID Okay. So, even when the patients were ready to come, who will attend to them?.’ (Nigeria [NGA], Program on Research for Vaccine Effectiveness [PROVE], Participant 03)

Another participant mentioned:

‘And there were also reports of like I said rejections from a lot of hospitals and of course, leading to some deaths and all that because services were severely, severely disrupted.’ (NGA/PROVE/Participant 01)

Participants also mentioned that some primary healthcare centres that are the closest to the people and entry point to healthcare systems in Nigeria shut down during the pandemic denying access to general and essential healthcare services like maternal and infant immunisation in some Nigerian communities. A respondent said:

‘A lot of hospitals, especially smaller ones, which is even nearer to people were all locked up, they were closed.’ (NGA/PROVE/Participant 13)

Another respondent mentioned:

‘You know for mothers or children, they are always at the primary health care centers. If it’s not immunisation, it is malaria you know, so with the fact that some of them shut down, I’m sure it really impacted negatively on them.’ (NGA/PROVE/Participant 10)

#### Sub-theme 1.2: Exposed health system gaps – inadequate PPEs

One of the factors that made HCWs stay away from the hospital or reject patients when the disease broke out in Nigeria was the dearth of personal protective equipment (PPE) in hospitals, which is required to attend to patients who need healthcare services during public health emergencies to prevent infection. A respondent mentioned:

‘And also the issue of PPE at the beginning, that was during the COVID, for the health workers, they were inadequate here and there, and everybody had children at home they wanted to protect themselves and also protect their families. So, there was this itch, a lot of things, everybody knows it was there, you know, our health system. We did not foresee all that, it affected the whole service delivery.’ (NGA/PROVE/Participant 05)

#### Sub-theme 1.3: Attention and resources shifted from other diseases to COVID-19

A substantial number of participants discussed that attention and resources were mainly focused on managing COVID-19 during the period, which affected timely response to other endemic diseases of importance that broke out about the same time as COVID-19 in Nigeria. A respondent mentioned:

‘Actually, other diseases of importance we didn’t have time for them. That was the time cholera was arriving, they were coming up. Because everybody went for COVID-19, so, many people were dying, but we were able to realise that look, this is our mandate. So we now try as much as possible to divide the team into two. If they’re going for COVID-19 in Oyo State and they are 8, we divide them into 4-4 so that they will able to take care of what is equally available.’ (NGA/PROVE/Participant 06)

#### Sub-theme 1.4: Disruption in the supply chain of essential drugs

Some participants also reported that some pharmacies were out of stock of some drugs as a result of the global restriction on international trade and movement during the pandemic, which affected supplies of drugs that are not produced locally in Nigeria. This blocked access to such drugs, especially among patients with non-communicable diseases who require such drugs to manage their health conditions. A respondent said:

‘You can recall that globally, there was a restriction on trades and travels. So, that also impacted negatively because some of the drugs that people were buying to manage maybe non-communicable diseases like hypertension or diabetes, they couldn’t get it again because they were imported. It affected people. Some die because the pharmacy that you need to go and buy these drugs are out of stock.’ (NGA/PROVE/Participant 02)

#### Sub-theme 1.5: Restriction of movement affected healthcare delivery and utilisation

It was also mentioned that restriction of movement as part of the strategies to contain the spread of the disease affected healthcare delivery and utilisation in Nigeria.

‘So, if I must, I must confess that COVID-19 had a lot of negative impact on service delivery and utilisation, of course, you know, then there was the restriction of movement at some point.’ (NGA/PROVE/Participant 02)

#### Sub-theme 1.6: Infection among healthcare workers disrupted services

It was reported that many HCWs contracted COVID-19 during the period, which affected service delivery in some departments and units of hospitals and had implications for patients who wanted to access such services. A respondent mentioned:

‘A lot that happened, a lot. And aside from that, of course, you know, even healthcare workers, many of them, had COVID, then, a lot of health workers became positive. And you know, the protocol was that you stay away from work. So you had situations whereby a whole department, all of them might be positive, and there was nobody to do the work. So the health patients didn’t have people to attend to them, and hospitals shut down sections of hospitals. So it affected it negatively.’ (NGA/PROVE/Participant 07)

#### Sub-theme 1.7: Fear of healthcare utilisation among patients

Even when healthcare services later became available during the pandemic, participants reported that patients were unwilling to utilise healthcare services because of the fear of contracting COVID-19 or being stigmatised as COVID-19 patients, especially when they show symptoms related to the virus. A respondent mentioned:

‘Some people were scared that, okay, they didn’t know who they were going to meet, so that one restricted some of them from visiting public facilities and others felt that it’s better to go there. So you can get good care depending on your level of enlightenment. Some people were actually scared. Because they don’t know who is who. Everybody was a suspect. Anytime anybody coughs or sneezes, you don’t know and you know it’s a droplet, it hangs in the air so, it’s two ways. Some people were discouraged from going to public facilities.’ (NGA/PROVE/Participant 04)

Another participant also said:

‘Oh!! during the COVID, during pandemic people were not, people were skeptical utilising it and all you know the implication now, if you get to, [*laughs*] you get to the facility you present with cough or catarrh and you, and you test positive, the stigmatisation was on the highest then, people were stigmatised even health workers were culprit to this stigmatisation, once they know that somebody is positive ahhh COVID ahhhh in fact people were afraid except it is highly essential, nobody wants to come near a patient.’ (NGA/PROVE/Participant 12)

### Theme 2: Positive impacts of COVID-19 on health service delivery and utilisation

Despite the negative impacts of COVID-19 on health service delivery and utilisation in Nigeria, most of the participants mentioned that healthcare systems in Nigeria received better attention during this period resulting in positive impacts on the healthcare sector. These positive impacts are organised into the following sub-themes.

#### Sub-theme 2.1: Improved public healthcare infrastructure

One of the frequently discussed themes on the positive impact of COVID-19 on the healthcare sector in Nigeria is that the pandemic resulted in improved public healthcare infrastructure for the country as the sector received increased attention from government, international and private individuals. One of the participants said:

‘As I said earlier, before the advent of COVID, the health system, and some of the infrastructure in the state had been dilapidated. So during the COVID, because it stimulated the government into action, a lot of interventions were received during that time at both individual level, governmental level, and even at this international level. And that one assisted the state to develop some of its infrastructure to meet some needs of the people. And after COVID, those infrastructures are still there today, serving even better than before the advent of COVID.’ (NGA/PROVE/Participant 08)

Another mentioned:

‘Okay, well, I will say that before that time, the health services has been okay. During that time, there was an improvement in health services delivery and that some hospitals actually they were upgraded, some infrastructure were upgraded, and there are some materials that were added even to some hospitals, you know, just to mention of it is this issue of oxygen generation, whereby oxygen concentrators were donated to some hospitals. And even presently, as we are talking now, we have some oxygen plant in the state ….’ (NGA/PROVE/Participant 11)

#### Sub-theme 2.2: Introduction of telemedicine in some hospitals

It was mentioned that the pandemic has informed the use of technology for service delivery in some Nigerian hospitals with the practice of telemedicine during the pandemic. A respondent said:

‘I think it has also somehow, you know, informed the use of telemedicine in a way because you have some facilities that I heard, you know, you can call in, and instead of going all the way. Now, because of that, the effect of lockdown, they could, they could prescribe, you could have some phone consultations, you can, you know, do some virtual meetings. And I think that’s one of the positive sides of the pandemic.’ (NGA/PROVE/Participant 03)

#### Sub-theme 2.3: Provided structure that can be leveraged for future health crises in some states

Participants also discussed that COVID-19 has provided a structure that can be leveraged to respond to other future disease outbreaks, primarily since the disease occurred on a large scale in Nigeria and triggered responses from different levels of government. For instance, a respondent discussed how the structures established in Oyo State can be used to respond to future outbreaks:

‘Let me say from the infrastructure, like I told you, up till three weeks ago, His Excellency also asked specifically, what about [*this Centre*]? I remember that when the former DG of Nigeria Centre for Disease Control, when he came to Oyo State and visited the [*the Centre*], he said, this is one of the best isolation/treatment Centers in the country. And I mean, so those structures are still there till today. And I remember what His Excellency said at that time was the fact that in case this pandemic goes down, what are you going to use these facilities for? But he insisted that [*the Centre*] should just be retained as such. And nobody knew that all these diphtheria and anthrax will come. And then Lassa fever and then other things will come … the place is ready to take any other person, people or be a center that we take, we use as a response center for infectious or public health diseases of importance.’ (NGA/PROVE/Participant 05)

#### Sub-theme 2.4: Readily trained health workforce for future health emergencies

It was also mentioned that considerable investment was made into training HCWs in planning for emergencies, responding, identifying and managing cases. Participants believed the skills acquired during the period provided HCWs with the required skills and knowledge to be deployed for future outbreaks:

‘Now, the Ministry of Health has a pool of people who are well-trained, these people were trained for almost one month or thereabouts. So, they have all the things, they need to know about planning for, responding, identifying, case management, everything they are, well, they’re prepared for that and I know this training also cascaded in some states. So right now, having that resource is one good thing that the country has done.’ (NGA/PROVE/Participant 01)

## Discussion

Findings from this study revealed that COVID-19 affected healthcare delivery and utilisation negatively during the early phase of the pandemic. The unwillingness of healthcare workers to deliver services emerged as the most discussed theme regarding the negative impact of COVID-19 on healthcare services. It was further observed that pre-existing health system gaps such as the inadequate supplies of PPEs, which are essential for the protection of healthcare workers in hospitals in critical health crises situations amplified the situation. This finding is similar to those of earlier studies conducted in six African countries, namely Zimbabwe, Malawi, Zambia, South Africa, Uganda and Botswana, where there was also a report of a lack of PPE in hospitals at the beginning of the pandemic.^28^ Another study also reported that most healthcare systems in low and middle-income countries were unprepared for the disease.^29^ This finding necessitates the need for increased supply of PPEs to healthcare institutions at different levels in Nigeria during both public health emergency and non-emergency periods. This will mitigate the fear of HCWs in delivering healthcare services to the public during such periods.

It was also mentioned that the attention of health stakeholders in Nigeria shifted away from other health conditions to COVID-19, neglecting other diseases of importance and delayed response to cholera that also broke out around the same period as COVID-19 in Nigeria. Similarly, in both high-income countries such as United Kingdom and Canada as well as low and middle income countries of the world such as Ghana, Uganda and Zimbabwe, disruptions to other healthcare services were also reported as attention shifted away from other diseases to COVID-19.^30,31,32,33^ However, considering the endemic nature of some parasitic diseases such as malaria and other chronic diseases of high morbidity and mortality in African countries like Nigeria, shift of attention from other diseases of importance during the pandemic in the country may have been associated with severe consequences when compared to the developed countries of the world. This finding implies that it is crucial for African countries to prioritise endemic diseases alongside new outbreaks, avoiding distraction by the urgency of novel outbreaks. This can be ensured by creating a robust surveillance system and response teams during novel outbreaks that will continue to keep track of both endemic diseases and novel outbreaks. This will require increased workforce with adequate knowledge, skills and resources. It is also important to create separate funds for novel outbreaks in addition to the existing funds budgeted for endemic diseases.

Previous studies have also reported that healthcare workers in some other countries, such as Sweden, contracted COVID-19, but with no negative effect on healthcare delivery.^34^ This was contrary to our findings, in which COVID-19 positivity rates among HCWs led to the shutdown of some health departments and units of some hospitals in Nigeria. This might have been associated with the shortage of healthcare staff in hospitals and the recent emigration of HCWs from Nigeria, leaving only a few healthcare professionals in the country’s hospitals who were also infected by the virus and the need to comply with COVID-19 protocols that involve proceeding on self-isolation upon testing positive. Poor infection and prevention control measures or inadequate PPEs within such hospitals might have also led to a high rate of infection among healthcare workers. This finding is similar to a study conducted in Kenya, which reported that healthcare workers went on strike during the pandemic because of increasing infection cases among healthcare workers during the period.^35^ It is therefore important that strong infection prevention and control measures be put in place in Nigerian hospitals such as hand hygiene facilities for patients and healthcare workers, ventilation systems, adequate PPEs and continuous education and training on infection prevention during public health emergencies for healthcare workers and issues of staff shortages in hospitals be addressed.

On the other hand, the social aspect of the disease, which was the public perception of hospitals as a hotspot for COVID-19 transmission, resulted in an unwillingness to utilise healthcare services. This was because of the fear of contracting COVID-19 or being diagnosed with COVID-19 when they show related symptoms, even when healthcare services later became available during the period. A similar experience was reported in Ghana by Aberese-Ako et al.^36^ They reported fear of utilising healthcare services in Ghana during the pandemic. To prevent future occurrences during public health emergencies, facilities that enhance infection prevention should be put in place across healthcare systems from primary to tertiary hospitals. This can bolster public confidence in healthcare systems during public health emergencies and change patient perception of hospitals as hotspots for disease transmission during public health crises which can encourage utilisation. It is also essential to introduce programmes that can help address the issue of stigma usually associated with novel disease outbreaks by highlighting stories of recovery using popular media platforms and also educating healthcare workers on sensitivity, empathy and non-judgemental care during such periods. This can encourage patients with related symptoms of such outbreaks to seek healthcare during such periods.

International and local movement restrictions which were reported to have affected healthcare utilisation in Nigeria, including the supply of essential drugs, were also reported in other African countries like Malawi.^37^ Nigeria and other African countries must therefore invest in building and enhancing the capacity of primary healthcare centres where patients can access healthcare services without necessarily going a long distance during public health emergencies and must also put in place conducive facilities and environment to encourage community health workers to live close by to such health facilities that can help to ensure uninterrupted service delivery during public health emergencies that may necessitate restricting movement which may affect transportation. Housing facilities should also be put in place for healthcare workers in Nigerian secondary and tertiary healthcare facilities to ensure the availability of healthcare staff in cases of patient referral from primary healthcare to secondary or tertiary level. Nigeria and other African countries need to also start working towards local production of essential drugs and vaccines through adequate investment in health research and training as well as providing infrastructures that can aid local production.

This study also found that COVID-19 positively impacted healthcare services in Nigeria despite the negative consequences of the disease. The positive impacts include an improved public health infrastructure as mentioned by a high majority of the participants, training of healthcare workers on emergency response and introduction of virtual consultation for service delivery, as well as structures that can be leveraged for future pandemics. The adoption of telemedicine in some Nigerian hospitals during the pandemic is worthy of note. This can improve healthcare devlivery and access during emergency and non-emergency periods for vulnerable individuals such as pregnant women, postpartum women and the elderly. It will be highly beneficial to further incorporate telemedicine into Nigerian healthcare systems across all levels. To achieve this, it is essential to invest in infrastructure that supports telemedicine in Nigerian hospitals and its utilisation in the community such as internet connectivity and electricity supply. Training and capacity building for healthcare workers is also crucial, which can improve virtual consultation skills of healthcare workers including awareness programmes to ensure a positive attitude towards its use among patients. It is also important for the country to develop a supportive policy framework with laws and regulations that guide telemedicine practice to ensure patient safety.

Previous studies have reported similar positive impacts of COVID-19 in Nigeria and Malawi.^37,38^ It is therefore important that the government at all levels in Nigeria maintain and sustain the improvement achieved in Nigerian public healthcare institutions during the pandemic period through increased budgetary allocations to the health sector. It is also essential that the government improves the working condition and welfare of the health workforce to curb their emigration to the Global North, which will help to retain the health workforce that the country has invested significant training and knowledge on public health emergency response into.

The limitation of this study is that information was not elicited from health services users to understand their individual and collective experiences and coping strategies during the pandemic. In addition, purposive sampling in this context risks selection bias, limits representativeness, relies on subjective criteria for informant selection, overemphasises elite opinions and may miss recent developments in healthcare. However, our study has some strengths that make our findings reliable. We have included key institutions and individuals with relevant oversight experience, enabling the collection of in-depth, targeted insights that are directly aligned with the study’s objectives. Future studies can explore patients perspectives and coping strategies during the pandemic especially among the vulnerable population such as those with chronic non-communicable and communicable health conditions and pregnant women. Preparedness of healthcare systems in responding to future outbreaks in both urban and rural communities of Nigeria and the extent of the implementation of practical solutions to strengthen their resilience can also be explored.

## Conclusion

During the early phase of the COVID-19 outbreak in Nigeria, healthcare service delivery was adversely affected. There was a shortage of PPEs in hospitals, leading to healthcare professionals being unwilling to provide services. Additionally, some primary healthcare centres were closed, limiting access to essential health services such as maternal and child health programmes. The focus on addressing COVID-19 also impacted the early response to a cholera outbreak, resulting in fatalities. Restriction of movement internationally and locally disrupted supplies of essential drugs, healthcare delivery and utilisation. The outbreak of COVID-19 among HCWs led to the closure of some units and departments in some hospitals affecting access to such healthcare services among those who needed it. The fear of contracting COVID-19 or being diagnosed with the disease also prevented the public who needed the services from accessing it. Despite these challenges, the pandemic presented an opportunity to address pre-existing deficiencies in the healthcare system. It is, therefore, important for the Nigerian government to maximise the gains from the pandemic to build a more resilient health system and devise strategies to mitigate the negative impact of future public health crises on healthcare service delivery and utilisation for non-emergency diseases. This can be achieved through health systems strengthening, which includes strengthening of primary healthcare systems through training of healthcare staff on health service delivery during public health emergencies, consistent supplies of adequate PPEs to healthcare institutions at all levels to enhance service delivery during both public health emergency and non-emergency periods, prioritising endemic diseases alongside new outbreaks by creating a robust surveillance system that detects and responds to endemic and novel outbreaks, creating separate funds for novel outbreaks without affecting existing funds budgeted for endemic diseases, putting in place facilities that enhance infection prevention across healthcare systems from primary to tertiary hospitals, which can bolster public confidence in healthcare systems during public health emergencies, introducing programmes that can reduce stigma associated with novel outbreaks, which can encourage healthcare utilisation and strengthening local capacity for drug and vaccine production.

It is also crucial that the government at all levels in Nigeria maintain and sustain the improvement achieved in Nigerian public healthcare institutions during the pandemic through increased budgetary allocations to the health sector for proper maintenance. It is also important that the government improves the working conditions of the health workforce to curb their emigration to the Global North, which will help to retain the health workforce that the country has trained for emergency periods.
